# Meeting of the American Medical Association

**Published:** 1864-04

**Authors:** 


### MEETING OF THE AMERICAN MEDICAL ASSOCIATION.

It will be observed that the meeting of the American Medical Association is to take place the 7th of June next, and is to be held in the city of New York. The profession will take a lively interest in the transactions of the Association. Measures of the greatest importance will be introduced for consider ation and urged for adoption. Medical Societies, Hospitals, and Medical Colleges, together with the Army and Navy should proceed at once to choose their delegates, furnish them with properly prepared certificates, and to notify the Committee of Arrangements, by sending a list of the names of their delegates.

This meeting will perhaps be the most important ever held by the Association, and members of the several Standing and Special Committees should be ready with full and suitable reports.

The profession cannot too highly estimate the opportunity afforded by this meeting, or be too early or active in preparing communications upon medical subjects to be presented to the Association. We would earnestly urge attention to this subject, and hope for immediate action.

